# Spatial–temporal characterization of rainfall in Pakistan during the past half-century (1961–2020)

**DOI:** 10.1038/s41598-021-86412-x

**Published:** 2021-03-25

**Authors:** Ghaffar Ali, Muhammad Sajjad, Shamsa Kanwal, Tingyin Xiao, Shoaib Khalid, Fariha Shoaib, Hafiza Nayab Gul

**Affiliations:** 1 College of Management, Shenzhen University, Nanhai Ave. 3688, Shenzhen, 518060 China; 2 Guy Carpenter Asia-Pacific Climate Impact Centre, School of Energy and Environment, City University of Hong Kong, Kowloon, Hong Kong, SAR; 3 Department of Civil and Environmental Engineering, Princeton University, Princeton, NJ 08544 USA; 4 Department of Land Surveying and Geo-Informatics, The Hong Kong Polytechnic University, Kowloon, Hong Kong, SAR; 5 Center for Policy Research On Energy and the Environment, Princeton University, Princeton, NJ 08544 USA; 6 Department of Geography, Government College University Faisalabad, Faisalabad, Pakistan; 7 School of Geographic Sciences, East China Normal University, Shanghai, China

**Keywords:** Climate-change policy, Environmental impact, Environmental impact

## Abstract

Spatial–temporal rainfall assessments are integral to climate/hydrological modeling, agricultural studies, and water resource planning and management. Herein, we evaluate spatial–temporal rainfall trends and patterns in Pakistan for 1961–2020 using nationwide data from 82 rainfall stations. To assess optimal spatial distribution and rainfall characterization, twenty-seven interpolation techniques from geo-statistical and deterministic categories were systematically compared, revealing that the empirical Bayesian kriging regression prediction (EBKRP) technique was best. Hence, EBKRP was used to produce and utilize the first normal annual rainfall map of Pakistan for evaluating spatial rainfall patterns. While the largest under-prediction was estimated for Hunza (− 51%), the highest and lowest rainfalls were estimated for Malam Jaba in Khyber Pakhtunkhwa province (~  1700 mm), and Nok-kundi in Balochistan province (~  50 mm), respectively. A gradual south-to-north increase in rainfall was spatially evident with an areal average of 455 mm, while long-term temporal rainfall evaluation showed a statistically significant (*p* = 0.05) downward trend for Sindh province. Additionally, downward inter-decadal regime shifts were detected for the Punjab and Sindh provinces (90% confidence). These results are expected to help inform environmental planning in Pakistan; moreover, the rainfall data produced using the optimal method has further implications in several aforementioned fields.

## Introduction

Rainfall is a key element of the Earth’s climate as well as being an uncontrollable natural phenomenon, and food security of developing agrarian nations highly depends on it^1–3^. Variability in rainfall patterns could significantly impact several aspects of ecosystems such as aquifer recharge, soil fertility, cropping patterns, and surface water resources, particularly in the rain-fed regions^3^. Moreover, rainfall is an important climate indicator, and its spatial–temporal assessment has significant implications^4,5^. For example, numerical assessments of the geographical heterogeneities and temporal trends of rainfall are integral to numerous purposes ranging from climate studies to runoff modeling, water resources management, agricultural research^6,7^, and risk assessment to natural hazards^8,9^. Thus, modeling the spatial–temporal distribution of rainfall and anomalous changes in its patterns can provide valuable, portable information that can be used in combination with other data sets to understand the impact of rainfall distribution and patterns on the society and environment^10,11^. Such improved understanding provides better opportunities to study manifold scientific problems such as aquifer recharge assessment and identification of drought–flood cycles. In this context, the need for continuous rainfall information in grid format is highly important for developing countries, particularly agrarian societies^12,13^, where vast applications are possible for irrigation scheduling and soil moisture modeling. However, the lack of information regarding rainfall distribution makes it difficult for communities in developing regions to take appropriate measures for ensuring their safety, livestock, and crop production. The prediction of rainfall, among several other hydro-meteorological parameters, is considered to be the most difficult to obtain because of its inherent spatial–temporal inconsistency^3–5,14^. This problem becomes increasingly complex in regions with diverse and complex topography^14^.

Although Pakistan, an autonomous state in South Asia, is situated between longitudes 60° E–75° E and latitudes 24° N–37° N (Supplementary Fig. S1), is often cited among the top ten countries likely to be affected by global warming^15^, existing research has mostly emphasized rainfall studies at the localized levels such as city, district, or provincial scales^4,11,16,17^, and none have presented a national-scale assessment. Even within these existing studies, spatial assessments are made using interpolation techniques following typical methods, rather than evaluating and adopting an optimal method.

Pakistan experiences an arid-to-humid climate with most rainfall in summer occurring due to monsoon phenomena and in winter due to western-disturbances such as extratropical storms from the Mediterranean Sea. Due to the lack of meteorological stations in most Pakistan cities, the availability of climate-related variables such as rainfall is challenging. Currently, climate data in Pakistan are only collected at specific meteorological stations, which hinders the availability of comprehensive data to government officials, institutes, and environmental and resource managers for different research purposes and decision making. To overcome this constraint, spatial interpolation techniques can be useful^18–20^. However, there is little, if any, consensus among researchers regarding the optimal interpolation method to estimate the continuous values of climate variables^21^; meaning that, a uniformly best possible method suitable for all climatic variables and data sets does not exist. However, a certain few preferred methods are typically utilized to produce the best representation of the distribution of different climatic variables over given regions. For instance, via evaluating the Thiessen polygon (THI), inverse distance weighting (IDW), ordinary kriging (OK) and universal kriging (UK) interpolation techniques^22^**,** found that THI method presents better results to interpolate rainfall in the Zayandeh Rud basin, Iran. On the other hand^23^**,** found that simple co-kriging technique is the most appropriate method to predict the rainfall distribution in the Hamadan province, Iran. Similarly^24^**,** identified that the principal component regression (PCR) is the best choice among IDW, PCR, and multiple linear regression to predict the rainfall distribution in Xinxie catchment area in southeastern China. The IDW has been identified as the best interpolation method by^25^ for the West Bengal, India. These variations in the optimal interpolation techniques for different regions highlight the need to determine the optimal interpolation technique for corrective spatial assessment.

In light of the above, in this study, we present a comprehensive spatial–temporal rainfall characterization of Pakistan for 1961–2020. For spatial assessment, we produced a normal rainfall map for Pakistan presenting the spatial distribution, as such information has been previously unavailable. For this purpose, the observed rainfall data collected at 82, out of total 97, countrywide stations (Supplementary Fig. S1) were retrieved, and several interpolation approaches (i.e., deterministic and geo-statistical) were comparatively analyzed to identify the best choice for the interpolation of rainfall in Pakistan. To compare interpolation techniques, an appropriateness index (AI) was computed based on multiple evaluation criteria, also known as cross-validation parameters, see Methods section “Comparing interpolation methods based on multiple cross-validation parameters”. The identified best method was then employed to produce a national-scale normal annual rainfall map of Pakistan using a spatial resolution of 11 km.

In addition to spatial assessments, the evaluation of rainfall on temporal scales is useful to understand the long- and short-term trends along with sudden regime-shifts in the rainfall, which could progressively help in agriculture, water resources management, and flood/drought-related decision-making^17,19,26–28^. For temporal analysis, we used the well-known Mann–Kendall test and Sen's slope estimator to detect long-term signals in the rainfall data. In addition, inter-decadal regime shifts (sudden change points), if any, were also evaluated using a Student’s t-test based algorithm^29^. The present study is novel in its evaluation of the best interpolation method for rainfall data and provides the first of its kind normal annual rainfall distribution map of Pakistan based on the optimal interpolation method. Being an agrarian society, while the gridded rainfall distribution and spatial evaluation provided here will have important implications for environmental planning and management (hydrological modeling, agricultural studies, climate assessments, water resources planning and management, and flood forecasting), the temporal rainfall trend assessment at the national and sub-national levels is equally important in the context of Pakistan’s climatic research.

## Data and methods

### Study area and data

In this study, rainfall data collected at 82 different meteorological stations randomly distributed throughout Pakistan were used, comprising annual average rainfall values (based on mean monthly rainfall data) covering the half-century period 1961–2020. The data used in this research were acquired and compiled from the Pakistan Meteorological Department, the Environmental Protection Agency in Pakistan, the Hydrocarbon Development Institute of Pakistan, and the Ministry of Climate Change, Pakistan. To reduce subjectivity, the skewness of the data was also analyzed, showing that overall data were positively skewed (coefficient = 3.562). To reduce this skewness, two commonly practiced methods, the log-transformation and Box–Cox transformation approaches, were applied for comparative transformation of the data^30^. Skewness was reduced by the log transformation to − 2.1337, and by the Box–Cox transformation to − 0.1484 (Supplementary Fig. S2); hence, the latter transformed data (λ = 0.29) was used for further analysis.

### Optimal interpolation method and spatial rainfall assessment

**Figure 1:**
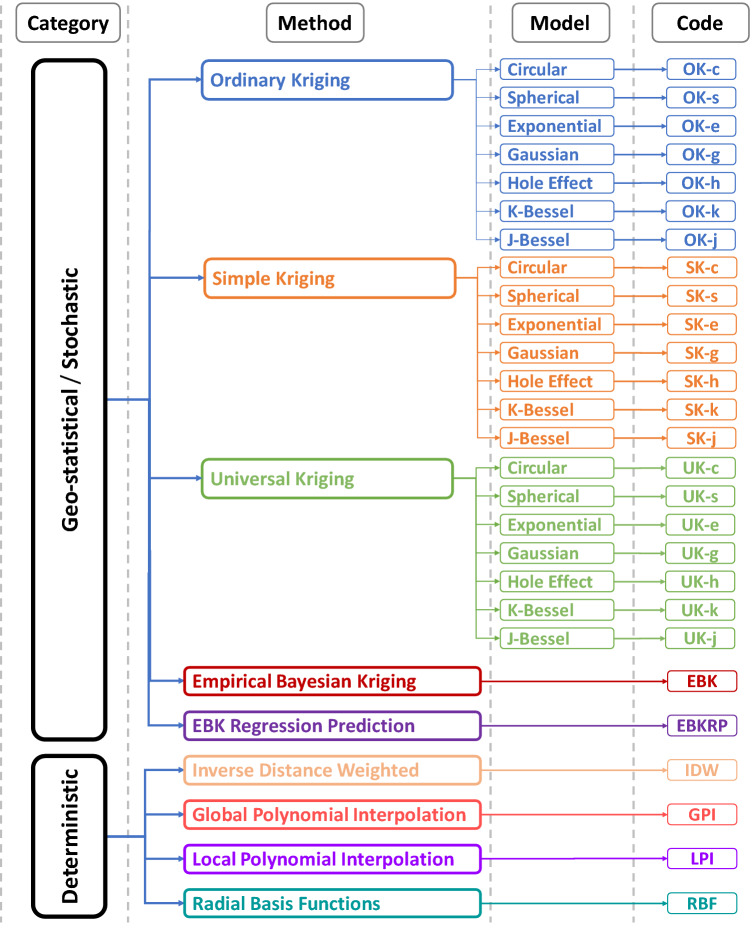
Schematic representation of different interpolation techniques selected for comparison. *The schematic is designed by a co-author M.S. in Microsoft Visio (Version 2013), * *available at* www.microsoft.com.

It is known that spatial interpolators can be data specific or sometimes even variable specific^31^. As the spatial interpolation outcomes are sensitive to the method’s dependency on the characteristics of available datasets, the selection of an accurate method and optimization of the interpolated values is a subjective yet critical step in generating accurate distribution maps of inherently continuous phenomena such as rainfall and temperature^32,33^. Given that various interpolation methods exist in the current literature for estimating unknown rainfall values, for this study, spatial interpolation methods were first systematically analyzed to assess the degree to which these different methods affect the unknown estimated rainfall values. Most of the existing studies in this field provided a limited comparison of interpolation methods, relying on only one or a few selected error statistics for selecting the optimal method. In this study, nearly all generally known interpolation methods were analyzed; further, different models within these methods were considered in order to discern the most optimal method for interpolating rainfall data in the study area. Based on a comprehensive literature review, we selected the interpolation techniques under two different categories (i.e., deterministic and stochastic)^21^. For the stochastic category, different semi-variogram models were also considered (i.e., Circular, Spherical, Exponential, Gaussian, Hole effect, K-Bessel, and J-Bessel), as they are known to significantly influence the prediction of unknown climate variable values. The codes for these methods are provided in Fig. 1, which will be used hereinafter. In this study, cross-validation focusing range parameter estimation was used to optimize the semi-variogram models and associated parameters such as nugget, sill, and range^21^. It should be noted that the area interpolation method was excluded from this comparison, as that method requires data assigned to polygons (areas) and our data were collected from individual stations^34^.

The deterministic category represents four methods, including inverse distance weighted (IDW), global polynomial interpolation (GPI), local polynomial interpolation (LPI), and radial basis functions (RBF). In contrast, the stochastic category presents five methods including ordinary kriging (OK), simple kriging (SK), universal kriging (UK), empirical Bayesian kriging (EBK), and empirical Bayesian kriging regression prediction (EBKRP). The common objective of all the selected interpolation methods is to estimate the unknown value of $\hat{Z}$ at the point $x_{0}$. This is given as:

1 $$\hat{Z}\left( {x_{0} } \right) = \mathop \sum \limits_{i = 1}^{n} \lambda_{i} Z\left( {x_{i} } \right)$$

where $Z\left( {x_{i} } \right)$ represents the measured value at point $x_{i}$, $n$ is the total number of existing data points, and $\lambda_{i}$ is the weight function allocated to data points^35^. The details of the different methods selected for the comparison are provided in the following sub-sections and can be found at: https://bit.ly/3k2Io5a.

#### Deterministic methods

*Inverse distance weighting (IDW) *is one of the most widely used, exact, and quick local interpolation methods. This method requires no subjective assumptions or pre-modelling in selecting the semi-variogram model, giving it advantage over other methods, particularly kriging. Under this method, the values are estimated using a linear combination of the value at the sampled point weighted by an inverse function of the distance between the two points. This method assumes that the observation points which are closer to the prediction points are more similar as compared to more distant points. We calculate the weights using:

2 $$\lambda_{i}^{IDW} = \frac{{1/d_{i}^{p} }}{{\mathop \sum \nolimits_{i = 1}^{n} 1/d_{i}^{p} }},$$

where $d_{i}$ represents the distance between the measured point $x_{i}$ and the predicted point $x_{0}$, *n* represents the total number of measured observations used, and $p$ represents a power parameter. The power parameter ($p$) defines the decrease in the weight as the distance increases. The power parameter for IDW used in our study was 3.41, after optimizing the model. The searching neighborhood shape is selected in such a way that all observations are used for the prediction.

*Global polynomial interpolation (GPI) *is a global, smooth (inexact), and deterministic trend-surface approach, ideal for addressing the need to make few decisions in the context of model parameters. This deterministic method has an underlying supposition that the fitting pattern in each predicted continuous surface represents underlying, gradually varying surface trends in the area. While the order of the polynomial defines the shape of the resultant surface, the first-order polynomial functions were used, as this gave a relatively large value (63) of the exploratory trend surface analysis (ETSA) after optimizing the model. The larger the value of ETSA, the more local the interpolation. Moreover, this method results in a smoothly varying surface using low-order polynomials. The simplest form of the first-order polynomial also known as linear polynomial is given as:

3 $$Z\left( {x_{i} , y_{i} } \right) = \beta_{0} + \beta_{1} x_{i} + \beta_{2} y_{i} + \varepsilon \left( {x_{i} , y_{i} } \right)$$

in which $Z\left( {x_{i} , y_{i} } \right)$ represents the datum at $\left( {x_{i} , y_{i} } \right)$ location, *β* the parameters, and $\varepsilon \left( {x_{i} , y_{i} } \right)$ represents random error.

*Local polynomial interpolation (LPI) *is a more flexible method than GPI, being a quick, local, and smooth trend-surface approach. Rather than adjusting a polynomial over the entire area, such as is done in GPI, the LPI adjusts different polynomials overlapping within a specified neighborhood. To make the comparison similar to the GPI, the first-order polynomial was used. This method is capable of capturing local variations in the data and can adapt to non-stationary and heterogeneous datasets. This local capturing is done using a moveable “window”—fitting local trends. The surface value, $\mu_{0} \left( {x_{i} , y_{i} } \right)$, at the center of this window is computed at each point. For first-order polynomial it can be represented as:

4 $$\mu_{0} \left( {x_{i} , y_{i} } \right) = \beta_{0} + \beta_{1} x_{i} + \beta_{2} y_{i} ,$$

and so on. The parameters *β* are estimated when the center point as well as the window moves in space.

*Radial basis function (RBF) *is a form of artificial neural network based on a structure that is three-layer feedforward (i.e., input-layer, hidden-layers, and output-layer)^36^. The interpolators covered by the RBF include a thin-plate spline, tension-based spline, regularized spline, and multi-quadric function. Splining can be visualized by fitting a bendable flexible rubber sheet through the sampled observations while minimizing the total curvature of the surface. All of these interpolators use a basic equation that depends on the distance between the measured and predicted points^37^. Under RBF, the predictor is a linear combination of the basis function, which is:

5 $$\hat{Z}_{RBF} \left( {x_{0} } \right) \approx \mathop \sum \limits_{i = 1}^{n} \lambda_{i} \varphi \left( {\left| {x_{i} - c_{i} } \right|} \right),$$

in which $\hat{Z}_{RBF} \left( {x_{0} } \right)$ is estimated using the sum of n radial basis functions $\varphi$ having different centers $c_{i}$ and weighted as per the coefficients $\lambda_{i}$.

#### Geo-statistical methods

*Simple kriging (SK) *is a flexible interpolator in nature and can be both smooth and exact. Therefore, it results in various surfaces such as probability, standard error, and prediction surfaces. SK is based on the kriging estimator:

6 $$\hat{Z}\left( {x_{0} } \right) - \mu \left( {x_{0} } \right) = \mathop \sum \limits_{i = 1}^{n} \lambda_{i} \left[ {Z\left( {x_{i} } \right) - \mu \left( {x_{i} } \right)} \right],$$

where $\lambda_{i}$ represents the kriging weight, n is the total measured points, $Z\left( {x_{i} } \right)$ is the measured variable value at a given data point $i$, and $\mu$ represents a known stationary mean, also called the trend component. $\mu$ is calculated as the mean of the data and is assumed to be constant over the entire study area. The weight $\lambda_{i}$ is derived using a semi-variogram or covariance function. In this study, a semi-variogram was used because of its wide application in different interpolation approaches, and was estimated using:

7 $$\gamma \left( {x_{i} x_{0} } \right) = \gamma \left( h \right) = \frac{1}{2}var\left[ {Z\left( {x_{i} } \right) - Z\left( {x_{0} } \right)} \right],$$

where $\gamma \left( h \right)$ represents the semi-variance and $h$ represents the distance between the measured and predicted data points. It should be noted that various semi-variogram models were used in this study to examine how selecting a different model influences the predictions. These models included circular, spherical, exponential, Gaussian, hole effect, K-Bessel, and J-Bessel. In SK, it is assumed that the trend component is an exactly known constant for the whole study area, which is approximated using the average value of the measured data, $\mu \left( {x_{0} } \right) = \mu$, such that:

8 $$\hat{Z}_{SK} \left( {x_{0} } \right) = \mu + \mathop \sum \limits_{i = 1}^{n} \lambda_{i}^{SK} \left( {x_{0} } \right) + \left[ {Z\left( {x_{i} } \right) - \mu } \right].$$

*Ordinary kriging (OK) *is also described as the acronym BLUE, representing “best linear unbiased estimator”^38^. It is given as:

9 $$\hat{Z}_{OK} \left( {x_{0} } \right) = \mathop \sum \limits_{i = 1}^{n} \lambda_{i}^{OK} \left( {x_{0} } \right)Z\left( {x_{i} } \right) with \mathop \sum \limits_{i = 1}^{n} \lambda_{i}^{OK} \left( {x_{0} } \right) = 1.$$

The only difference between OK and SK is that in OK, $\mu$ (the unknown trend constant) needs to be approximated. One of the key considerations of OK is that it assumes that the mean values remain constant over the whole are to be interpolated.

*Universal kriging (UK)* is also known as regression kriging, kriging with a trend, and external drift-based kriging. UK is also known as a multivariate extension of OK, which uses a higher deterministic or linear trend function $\mu \left( {x_{i} } \right)$ rather than relying on a constant trend function $\mu$. In this case, the local trend function is given as:

10 $$\mu \left( {x_{0} } \right) = \mu \left( {x, y} \right) = a_{0} + a_{1} x + a_{2} y.$$

It should be noted that an exponential kernel function was used in this study as a trend function, as it results in the most satisfactory results^21^.

*Empirical Bayesian kriging (EBK) *is a robust and straightforward interpolation technique that requires minimal interactive modeling. In this geo-statistical interpolation technique, the most difficult aspects of a kriging model building are automated. This implies that, rather than adjusting the parameters to receive accurate results manually, the EBK computes the parameters automatically using a sub-setting and simulation procedure. The key difference between EBK and other kriging approaches is that EBK accounts for the errors caused by the estimation of the underlying semi-variogram. The other kriging approaches underestimate the prediction standard errors because they do not account for the semi-variogram’s uncertainty. It is noted that EBK is more of an algorithm based on 6 different steps (see Gribov and Krivoruchko 2019 for further details) designed to automate the most difficult aspects of building a valid kriging model through a process of sub-setting and simulations. It is based on two different geo-statistical models (i.e., linear mixed model and intrinsic random function kriging). Readers are encouraged to see^36^ for further details on this.

*Empirical Bayesian kriging regression prediction (EBKRP) *is a relatively new geo-statistical interpolation approach. It is an advanced form of EBK and considers different additional explanatory indicators (in raster format) that are known to influence the estimation of the dependent variable—acting as prior information. In this approach, regression analysis is coupled with the kriging method to make the interpolations more precise than the ones estimated by kriging and regression alone. This approach is a hybrid interpolation method that uses simple kriging (Eq. 4) and ordinary least squares (OLS) regression. The dependent variable (rainfall in this case) is approximated based on kriging and regression models by separating the estimation of the average variable value and an error term. This can be expressed as $Dependent variable = avarage + error$. Here, the difference between OLS and kriging approach is that the main emphasize in OLS is to model *average* whereas, kriging models *error* through a semi-variogram. In EBKRP, both regression and a semi-variogram models for average and error term, respectively, are modeled simultaneously. This simultaneous operation on both terms results in more precise prediction of the variable as compared with individual application. In this study, elevation data was used as an explanatory variable due to its influence on rainfall. Further, a 30-m spatial resolution digital elevation model-based raster was used as an input parameter for the dependent variable.

### Comparing interpolation methods based on multiple cross-validation parameters

Different parameters of cross-validation (a well-known statistical approach to assess the preciseness of the interpolated data) were used to evaluate the performance of the different interpolation methods. In cross-validation, the performance of interpolation is determined by iteratively removing an observed value from the dataset and re-estimating it using the remaining values. An error is determined by estimating the difference between each observed (measured) and predicted value. The cross-validation statistics performed on these errors are expected to be reasonable evaluation estimators for comparing the different interpolation models and forming the basis of the method selection process. Based on extensive literature review, we focus on six cross-validation methods: mean error (ME), root mean square error (RMSE), Pearson R^2^ (R2), mean standardized error (MSE), root mean square standardized error (RMSSE), and average standard error (ASE)^17–21,39,40^. Few studies have included all six cross-validation parameters to determine the optimal interpolation technique due to the complexity and higher likelihood of human error. Most studies have used only one or two parameters to determine the most suitable interpolation technique, with RMSE and R2 being the most widely used^30,41,44^.

To facilitate this selection process, an appropriateness index (AI) was computed based on the underlying cross-validation indicator/parameters and their contribution towards the suitability of a certain interpolation method. These validation methods are considered to be positively contributing if the increase in the indicator value results in the interpolation method being more appropriate than others, and vice versa. Among these parameters, an increase in the parameter value (for all parameters except R^2^) indicates that the interpolation technique is less suitable. To compute the AI, the values of all the parameters were normalized to non-dimensional using a minimum–maximum normalization method^45–47^. For the normalization of ME, RMSSE, ASE, RMSE, and MSE, the following was used:

11 $$\left\{ {X_{j } {-}min(X_{j} } \right\}/\left\{ {max\left( {X_{j} } \right){-}min\left( {X_{j} } \right)} \right\},$$

due to their negative contribution towards the suitability. For R2, the following was used:

12 $$\left\{ {max\left( {X_{j} } \right) - X_{j} } \right\}/\left\{ {max\left( {X_{j} } \right){-}min\left( {X_{j} } \right)} \right\},$$

because of its positive contribution towards suitability. Here, *X*_*j*_ represents the parameter value for the interpolation method *j.* This approach helps to distribute the values of each parameter between 1 and 0, making them non-dimensional, where a value closer to 1 indicates that the technique is more suitable, and vice versa. Based on these normalized values of the cross-validation parameters, the AI was computed using the following equation:

13 $$AI_{{}} = \left( {\mathop \prod \limits_{i = 1}^{n} R_{i} } \right)^{1/n} ,$$

where *AI* is the appropriateness index, *R* is the normalized value of the cross-validation parameter for interpolation technique *i*, and *n* is the total number of parameters considered for the computation of the AI. It should be noted that because the EBK and EBKRP were identified as the most optimal methods, a continuous ranked probability score (CRPS) was further considered, as this score is only available for these two methods in ArcGIS. The CRPS is known to be a good evaluating scoring rule of probabilistic forecasts in the context of a univariate-quantity^39^. The CRPS is oriented negatively, implying that, the smaller the values, the better the performance of the method. It is calculated as:

14 $$CRPS\left( {P, x} \right) = \mathop \smallint \limits_{ - \infty }^{\infty } \left( {P(y} \right) - I\left( {y \ge x} \right))^{2} dy,$$

where $P$ represents the cumulative distribution function of the density forecast and $x$ represents the observed rainfall (normalized). The values of CRPS inside 90% and 95% confidence intervals were used to compare the methods. The details of all cross-validation parameters are presented in Table 1.

**Table 1:** Details on multiple cross-validation parameters used to compute the appropriateness index (AI).

Cross-validation parameter	Description	Formula
Mean Error (ME)	The Mean Error (ME) represents the arithmetic average of all the estimated errors in the interpolation. It also tells the direction (average) of the estimated errors. The positive bias represents an overestimation of the variable whereas the under-estimation is represented by negative bias. This parameter is used as an accuracy indicator because positive and negative estimations counteract each other resulting the ME comparatively lower than the actual error^40^	${\text{ME}} = \frac{1}{N}\mathop \sum \limits_{i = 1}^{N} \left[ {\overline{P}_{i} - \overline{O}_{i} } \right]$
Root mean square error (RMSE)	The Root mean square error (RMSE) is the most widely and commonly used cross-validation parameter. It holds the same measuring unit as the predicted value (e.g. inches)	${\text{RMSE}} = \sqrt {\frac{1}{N}\mathop \sum \limits_{i = 1}^{N} \left[ {\overline{P}_{i} - \overline{O}_{i} } \right]^{2} }$
Root mean square standardized error (RMSSE)	It is ideal to have the Root mean square standardized error (RMSSE) close to one. If the RMSSE > 1, then it indicates a general under-estimation in the inconsistency of the estimated/predicted variable. If the RMSSE < 1, then it indicates a general overestimation in the inconsistency of the estimated/predicted variable	${\text{RMSSE}} = \sqrt {\frac{{\mathop \sum \nolimits_{i = 1}^{N} \left( {\frac{{\overline{P}_{i} - \overline{O}_{i} }}{{\sigma \left( {\overline{O}_{i} } \right)}}} \right)^{2} }}{N}}$
Average standard error (ASE)	Average Standard Error (ASE) measures the arithmetic average of the prediction standard errors. It represents the error magnitude showing the method’s accuracy	${\text{ASE}} = \sqrt {\frac{{\mathop \sum \nolimits_{i = 1}^{N} \sigma^{2} }}{N}}$
Mean standardized error (MSE)	Mean Standardized Error (MSE) provides the average of the standardized errors. The value of MSE is better if it is close to 0	${\text{MSE}} = \frac{{\left( {\mathop \sum \nolimits_{i = 1}^{N} \frac{{(P_{i} - O_{i} )}}{{\sigma { }}}} \right)}}{N}$
Pearson R^2^ (R2)	It measures the correlation (linear) between the measured and the predicted covariance (rescaled). The resultant values range between − 1 and 1 where the value closer to − 1 indicates a strong negative correlation and the value closer to 1 indicates a strong positive correlation. The value closer to zero represents a week correlation The Pearson R^2^ is widely used to measure the goodness of fit for different interpolation methods. However, as noted by Li and Heap, it could often be misleading and hence, one should be careful in using this single parameter as an evaluation criterion	$R^{2} = \frac{{\left[ {\mathop \sum \nolimits_{i = 1}^{N} (P_{i} - \overline{P}_{i} } \right)\left( {O_{i} - \overline{O}_{i} } \right)]^{2} }}{{\mathop \sum \nolimits_{i = 1}^{N} (P_{i} - \overline{P}_{i} )^{2} \mathop \sum \nolimits_{i = 1}^{N} (O_{i} - \overline{O}_{i} )^{2} }}$
$O_{i}$ represents observed/measured value $P_{i}$ is the predicted value *i* is where predicted value is to be estimated $\overline{O}_{{}}$ is the average of observed/measured values $\overline{P}_{{}}$ is the average of predicted values		$\sigma$ is the inconsistency/variation in the dataset also explained as the standard deviation of the estimation error $N$ represents the total number of observed/measured values

### Temporal analysis of rainfall in Pakistan

To analyze the temporal rainfall trends in Pakistan, the well-known Mann–Kendall test and Sen’s Slope estimator were selected for long-term assessment^46^. The Mann–Kendall test is given by:

15 $$VRS\left( S \right) = \frac{1}{18}\left[ {n\left( {n - 1} \right)\left( {2n + 5} \right) - \mathop \sum \limits_{p = 1}^{q} t_{{p{ }}} \left( {t_{p} - 1} \right)\left( {2t_{p} + 5} \right)} \right],$$

where $q$ is the number of tied groups and $t_{p }$ is the number of data values in the “pth” group. The values of $S$ and $VRS\left( S \right)$ were used to compute the test statistic *“Z”* as follows:

16 $$Z = \left\{ {\begin{array}{*{20}l} {\frac{{S - 1}}{{\sqrt {VAR\left( S \right)} }}} \hfill & {if\,\,S > 0} \hfill \\ 0 \hfill & {if\,\,S = 0} \hfill \\ {\frac{{S + 1}}{{\sqrt {VAR\left( S \right)} }}} \hfill & {if\,\,S < 0} \hfill \\ \end{array} } \right..$$

The two-tailed test at 0.001, 0.01, 0.05 and 0.1 levels of significance was used to detect a positive or negative value of “Z.” The null hypothesis was rejected if the absolute value of *“Z”* was greater than Z_1-α/2_, where Z_1-α/2_ was obtained from the standard normal cumulative distribution tables.

Further, Sen’s non-parametric method was used to estimate the true slope of an existing trend as change per unit of time (year, in this case). Once the trend was determined, Sen’s slope estimation was used to calculate the magnitude of the trend slope, which is:

17 $$f\left( t \right) = {\mathcal{Q}}t + B,$$

where *t* refers to the year, *Q* refers to the trend slope (the tendency is more obvious when Q is greater), and *B* is constant.

Additionally, a regime shift detection algorithm was applied to assess the inter-decadal rainfall trends in Pakistan^48,49^. This algorithm is based on the Student’s t-test, which is sensitive to deviation in the successive running averages of the variable values under a given cut-off length (10 years, in this case). These regimes shift (inter-decadal variations) were analyzed with 90% confidence. Both the long- and short-term evaluations were conducted at national and sub-national (provincial) levels in order to provide more detailed insights.

## Results and discussion

### Optimal interpolation method for rainfall interpolation in Pakistan

The statistics of cross-validation and the results on the AI are presented in Table 2. A two-step evaluation approach was used to select the most optimal interpolation technique. In the first step, the AI was computed based only on three cross-validation parameters (i.e., MR, RMSE, and Pearson R^2^, represented by AI-3 in Table 2), as these parameters were available for all possible interpolation techniques including the IDW, GPI, and RBF. Based on the ranking of all interpolation methods, it was evident that none of the typical techniques were optimal for Pakistan. Based on the AI-3 ranking, the EBKRP method was identified as the best choice with an AI-3 value of 1. The order of suitability of the interpolation techniques according to AI-3 was EBKRP > UK-k > EBK > IDW > OK-k > OK-e > RBF > OK-g > OK-s > OK-c > OK-j > UK-h > UK-e > SK-k > UK-j > SK-e > OK-h > SK-s > UK-c > UK-s > UK-g > LPI > SK-c > SK-g > SK-h > SK-j > GPI. This indicates that if only the ME, RMSE, and R2 parameters of the cross-validation are considered, the IDW would perform best among all the deterministic interpolation techniques with an AI-3 value of 0.94 and an overall rank of 4. However, it would not perform as well compared to the geo-statistical and stochastic interpolation techniques.

**Table 2:** Cross-validation statistics from all interpolation methods and the appropriateness index (AI). AI-3 represents the first step of evaluation whereas AI-6 represents the second step of evaluation of the optimal interpolation technique. The values of cross-validation parameters are normalized using the minimum–maximum normalization method as discussed in section “Deterministic methods”.

Method	ME	RMSE	R2	MSE	RMSSE	ASE	AI-3	Rank AI-3	AI-6	Rank AI-6
IDW	0.9428	0.9351	0.9494	–	–	–	0.9430	4	–	–
GPI	0.9464	0.0000	0.0000	–	–	–	0.0000	26	–	–
RBF	0.7973	1.0000	1.0000	–	–	–	0.9280	7	–	–
LPI	0.4499	0.7589	0.8121	0.3564	0.9890	0.5263	0.6549	22	0.5524	15
EBK	0.9280	0.9527	0.9628	1.0000	1.0000	0.8530	0.9482	3	0.9379	2
OK-c	0.8314	0.9305	0.9456	0.8510	0.4728	0.6274	0.9020	10	0.7133	6
OK-s	0.8157	0.9432	0.9561	0.8261	0.4652	0.6252	0.9036	9	0.7070	7
OK-e	0.8716	0.9604	0.9691	0.8973	0.3678	0.5549	0.9333	6	0.6829	8
OK-g	0.8789	0.9202	0.9375	0.8855	0.4717	0.6004	0.9127	8	0.7174	5
OK-h	0.4922	0.9007	0.9253	0.4863	0.1819	0.3240	0.7452	17	0.4112	22
OK-k	0.9204	0.9338	0.9476	0.9476	0.4845	0.6328	0.9345	5	0.7495	3
OK-j	0.8387	0.9153	0.9343	0.8433	0.6094	0.6058	0.8961	11	0.7409	4
SK-c	0.3122	0.8316	0.8649	0.3494	0.6906	0.6766	0.6108	23	0.5162	18
SK-s	0.5312	0.8343	0.8661	0.5807	0.4865	0.5290	0.7291	18	0.5646	12
SK-e	0.5135	0.8825	0.9066	0.5601	0.6091	0.6571	0.7456	16	0.6206	9
SK-g	0.3140	0.8200	0.8552	0.3478	0.7249	0.6734	0.6069	24	0.5182	17
SK-h	0.2878	0.7573	0.8004	0.3486	0.5651	0.4512	0.5620	25	0.4345	20
SK-k	0.6010	0.8766	0.9013	0.6439	0.4888	0.5864	0.7821	14	0.6145	10
SK-j	0.0000	0.7974	0.8376	0.0000	0.7438	0.6565	0.0000	26	0.0000	23
UK-c	0.4652	0.8786	0.9071	0.5163	0.2183	0.3245	0.7208	19	0.4230	21
UK-s	0.4502	0.8889	0.9136	0.5110	0.3055	0.4062	0.7174	20	0.4710	19
UK-e	0.5655	0.9206	0.9379	0.6694	0.3469	0.4569	0.7893	13	0.5531	14
UK-g	0.4380	0.8543	0.8832	0.5552	0.4937	0.4571	0.6939	21	0.5289	16
UK-h	0.6553	0.8679	0.8943	0.9088	0.0000	0.0000	0.8000	12	0.0000	23
UK-k	1.0000	0.9219	0.9429	0.9932	0.1674	0.3701	0.9548	2	0.5567	13
UK-j	0.5679	0.8788	0.9032	0.6843	0.4948	0.4965	0.7688	15	0.5969	11
**EBKRP**	**0.9629**	**0.9558**	**0.9653**	**0.9150**	**0.9364**	**0.9774**	**0.9617**	**1**	**0.9425**	**1**

Given that IDW, GPI, and RBF were not suitable interpolation techniques for the rainfall data in Pakistan, the AI was re-computed based on all available parameters of the cross-validations (six, i.e., ME, RMSE, R2, MSE, RMSSE, and ASE), termed as AI-6 in this study. AI-6 also indicated the EBKRP (AI-6 = 0.9425) as the most appropriate interpolation technique followed by EBK (AI = 0.9379). The order of suitability of the interpolation techniques according to AI-6 was EBKRP > EBK > OK-k > OK-j > OK-g > OK-c > OK-s > OK-e > SK-e > SK-k > UK-j > SK-s > UK-k > UK-e > LPI > UK-g > SK-g > SK-c > UK-s > SK-h > UK-c > OK-h > SK-j > UK-h. Results from both AI-3 and AI-6 show that among all kriging approaches, the ordinary kriging-based techniques performed comparatively well. As EBK and EBKRP were the top two interpolation methods for interpolation based on AI-6, we further compared them on the basis of CRPS using confidence intervals of 90% and 95%. The CRPS values for EBK and EBKRP in the 90% confidence interval were 91.46 and 89.02, respectively. Similarly, the CRPS values for EBK and EBKRP in the 95% confidence interval were 93.90 and 92.68, respectively. Because the CRPS is negatively oriented, smaller values correspond to better methods. Hence, CRPS also showed that EBKRP is the most optimal technique for interpolating rainfall in Pakistan, as the values of CRPS for EBKRP under both confidence intervals (i.e., 90% and 95%) were smaller than those of EBK.

**Figure 2:**
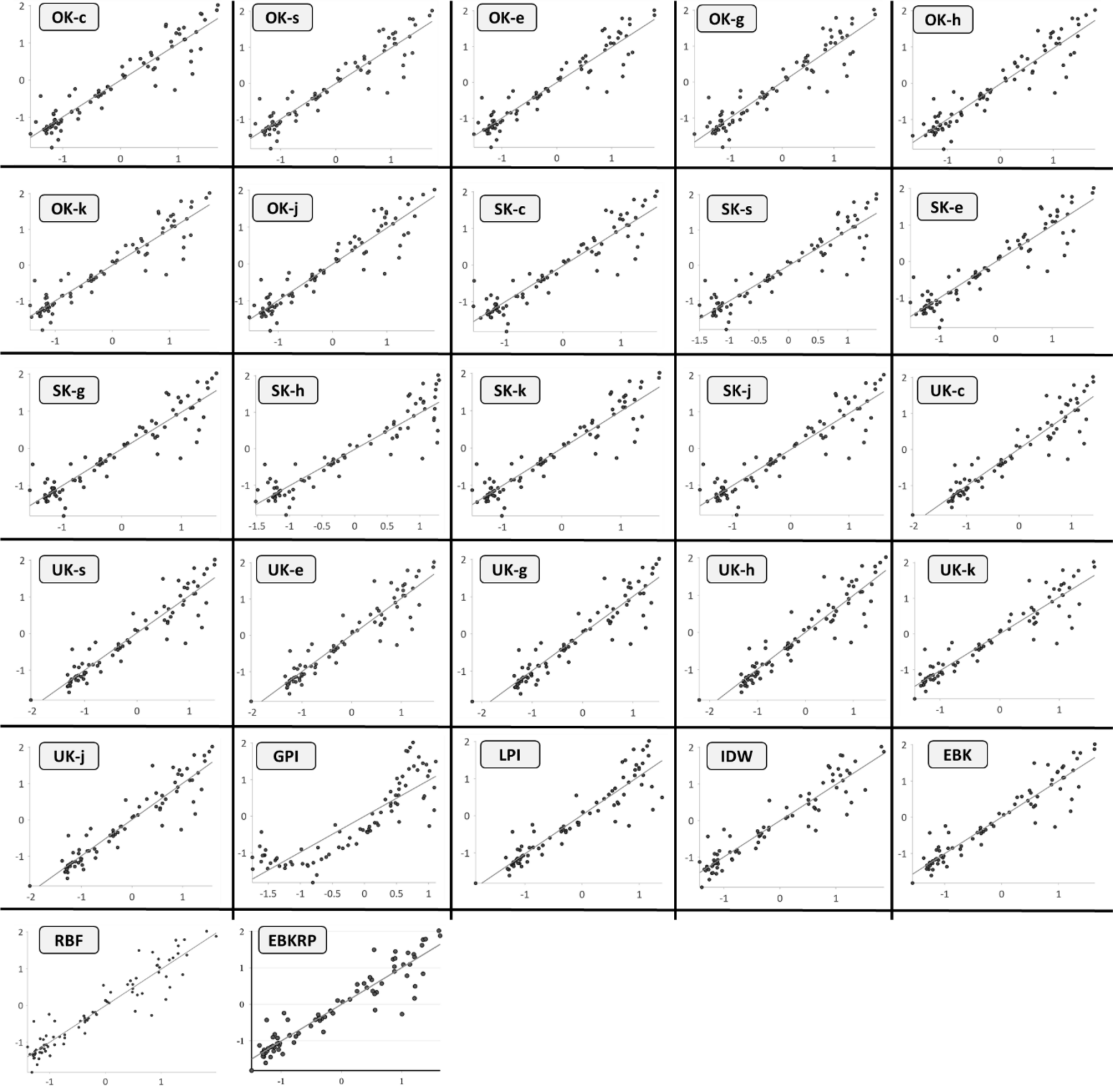
Scatter plots of measured (y-axis) vs. predicted (x-axis) rainfall values from all interpolation methods. *The plots are generated in ArcGIS Pro (Version 2.7) from the Environmental Systems Research Institute (ESRI) by a co-author M.S., * *available at* www.esri.com.

The scatter-plots for measured vs. predicted rainfall show inconsistencies among the different interpolation methods used to interpolate the rainfall in the study area (Fig. 2). This inconsistency also existed for a single interpolation method when different semi-variogram models (i.e., spherical, exponential, circular, Gaussian, hole-effect, k-Bessel, and j-Bessel) were employed, as shown in Fig. 2. Hence, care should be taken in selecting not only the interpolation method but also the semi-variogram model to determine the optimal interpolation method. Notably, most points where rainfall prediction was under-estimated were located in the northern regions of Pakistan. This underestimation could possibly be a result of the complex physiography of that region (i.e., mountainous terrain), making the estimation more difficult in comparison to the plain regions such as the southern Punjab and Sindh provinces. Using the scatter-plot for the EBKRP interpolation method, it was estimated that the largest under-estimation in predicted rainfall was for Hunza station (− 55%) in northern Pakistan (Supplementary Fig. S1).

The optimal interpolation technique for rainfall in Pakistan identified in our study is different from that of^41^, who concluded that the universal kriging with hole effect model (UK-h) is best for the Indian Himalayas^16^. One possible reason for this different result could be the used comparison approach along with the scale of their study, which was substantially smaller than that of the present study. Overall, the identified optimal interpolation method from this study is important for Pakistan and could be adopted by institutions, researchers, and professionals responsible for national-level rainfall data archiving and inventory management (i.e., Pakistan Meteorological Department) to support the consistency of rainfall data. This empirically validated interpolation technique can result in more reliable rainfall information as compared with typical in-practice interpolation methods such as IWD or simple kriging, which can ultimately be utilized in further research.

### Spatial distribution of rainfall in Pakistan

**Figure 3:**
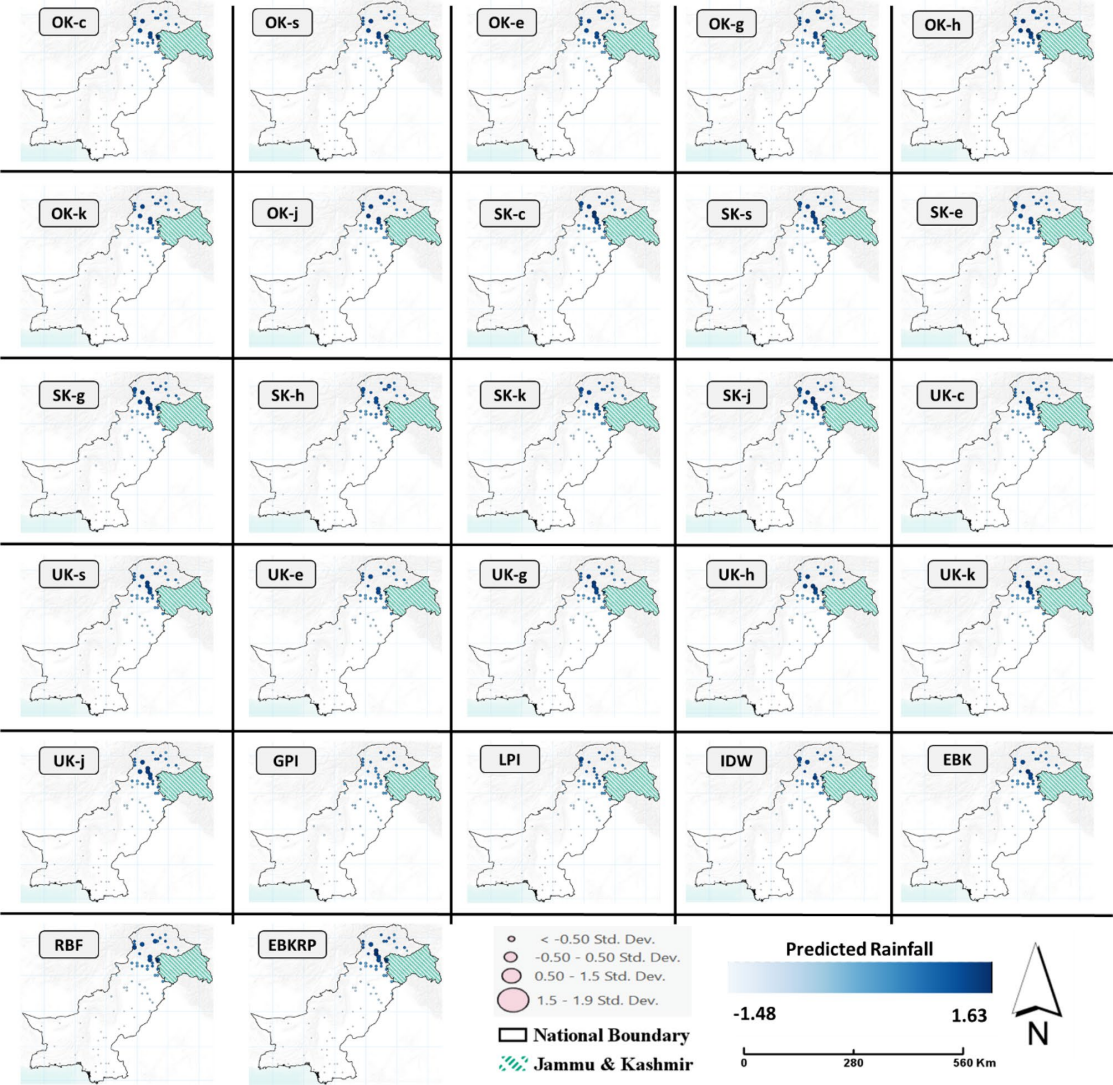
Predicted rainfall at all observation stations from different spatial interpolation methods. The size of the circles is based on the standard deviation showing how much the predicted rainfall values diverge (i.e., above or below) from the overall mean. All rainfall values are Box-Cox transformed for normality. *The maps are designed using ArcGIS Pro (Version 2.7) from the Environmental Systems Research Institute (ESRI) by a co-author M.S., * *available at* www.esri.com.

**Figure 4:**
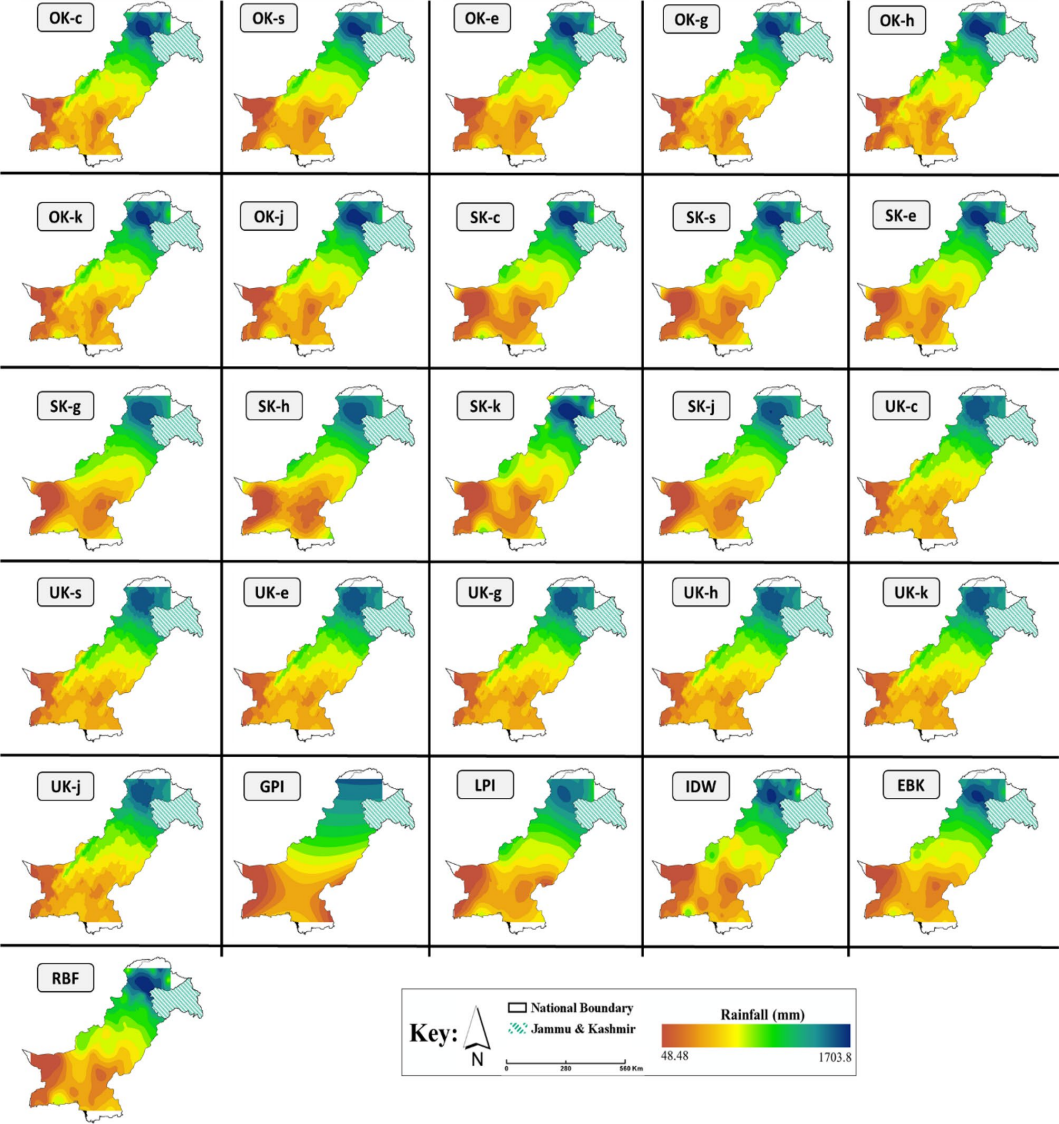
Spatial distribution of normal annual rainfall in Pakistan based on different interpolation methods. *The maps are designed using ArcGIS Pro (Version 2.7) from the Environmental Systems Research Institute (ESRI) by a co-author M.S., * *available at* www.esri.com.

**Figure 5:**
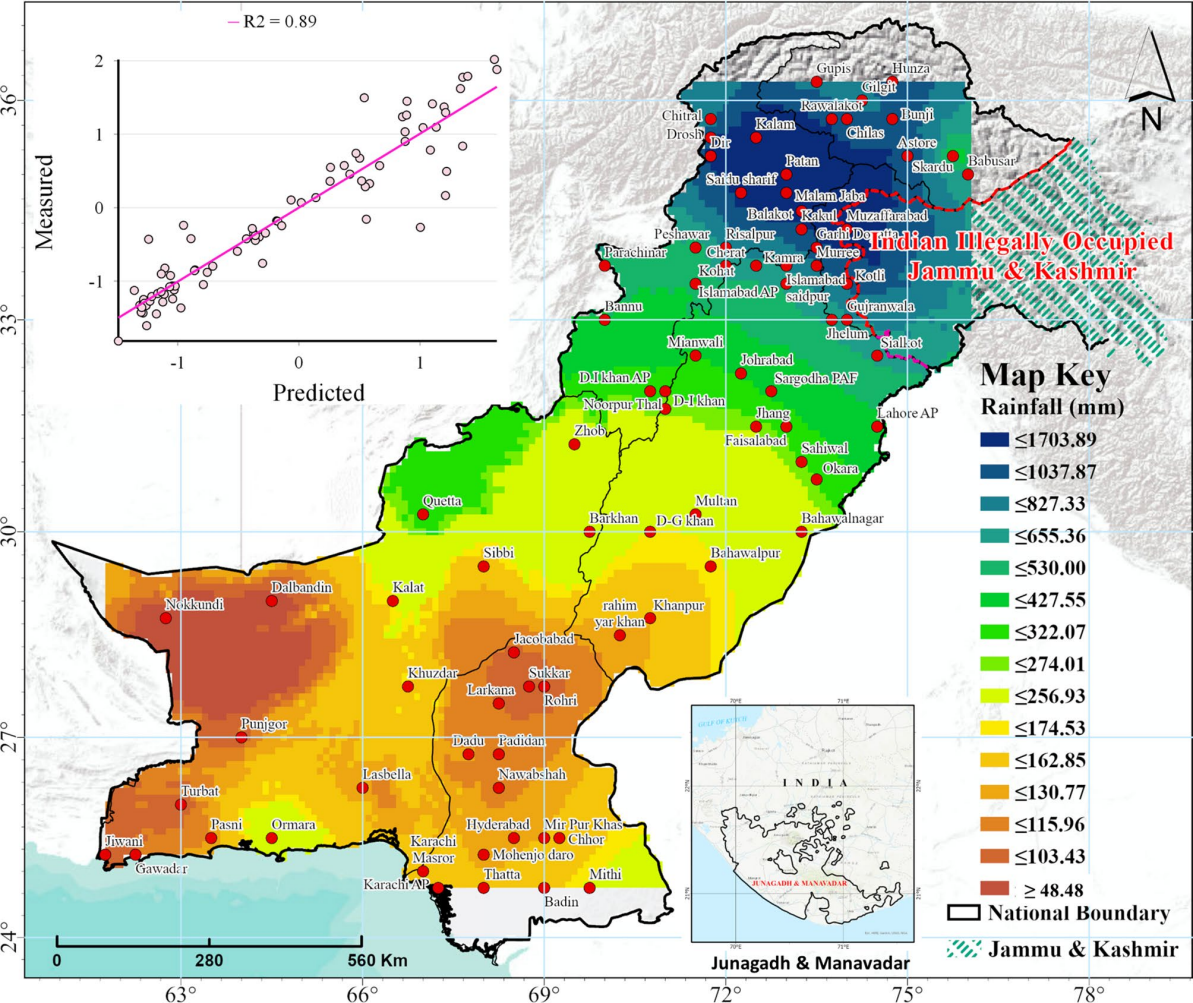
Normal rainfall map of Pakistan based on the empirical Bayesian kriging regression prediction (EBKRP) method for interpolation; the inset scatter-plot shows the measured vs. predicted values. The pink line shows the R2 for the predicted values. The shades in brown represent drier areas (relatively less rainfall) and green-to-blue shades show comparatively wet areas (relatively more rainfall). Red dots indicate the observation stations (n = 82). *The map is designed using ArcGIS Pro (Version 2.7) from the Environmental Systems Research Institute (ESRI) by a co-author M.S., * *available at* www.esri.com.

The predicted rainfall at all observation stations using different interpolation methods showed clear spatial heterogeneity throughout the study area. Most of the stations with comparatively larger rainfall (std. dev. > 1.5) belonged to the Khyber Pakhtunkhwa (KPK) province and Gilgit Baltistan region (Fig. 3). The final normal rainfall map of Pakistan using the EBKRP method showed clear spatial variability in the rainfall from the south to the north (Fig. 3). Almost all the interpolation methods showed a similar general distribution and pattern of the rainfall in Pakistan; indicating an increase in the rainfall from south to north (Fig. 4). However, spatial variations among different interpolation methods were evident with a clear difference between the deterministic and stochastic methods (e.g., see the spatial demarcation of brown, green, and blue shades in Fig. 4). Based on the optimal interpolation method of EBKRP, the estimated normal annual rainfall in Pakistan ranged from ~ 50 to ~ 1700 mm throughout the study area with an areal average rainfall of 455 mm (Fig. 5). From a cross-regional perspective, the lowest rainfall (ranging from 48–48 to 162.85 mm) in the study area was experienced by the south-western areas in Balochistan province and the central region in Sindh province. Results indicate that the lowest rainfall was in Nok-kundi, Balochistan (~ 50 mm, within 95% confidence interval).

The northwestern regions of Balochistan (i.e., Quetta) experienced comparatively larger rainfall (presented as green shades) than southern Balochistan and most of Sindh province (presented as brown shades in Fig. 5). One possible reason for the larger rainfall in the Quetta region could be its higher altitude compared to other southern regions (Fig. 1). However, further assessment in this regard is needed to analyze the correlation between altitude and rainfall occurrence. This could be a useful research question in the context of Pakistan to further improve our understanding of local spatial inconsistencies in rainfall patterns. The inner-Sindh province includes the areas of Rohri, Jacobabad, Sukkar, Larkana, Nawabshah, and Dadu, which were among the lowest rainfall areas. This region is highly water-scarce, thus the lower rainfall coupled with increasing population and decreasing per-capita water capacity could result in worsening consequences without proper planning and management^1,50,51^. To address this, the normal rainfall data produced in this study could assist in this regard to effectively improve water resources planning and management for Pakistan, which is among the top fifteen countries facing extremely high baseline water stress^32^. According to the results, most regions in Punjab experienced moderate to high rainfall ranging from ~ 275 to 830 mm, with southern Punjab experiencing lower rainfall compared to northern Punjab (Fig. 4). The highest rainfall in Pakistan was experienced by the northern regions of KPK and Gilgit Baltistan (~ 1000–1700 mm). Among all the regions, the highest rainfall was estimated for the Malam Jabba region in KPK province (~ 1700 mm). This finding is in agreement with the results of^4^ who presented a localized rainfall assessment in Pakistan’s northwestern regions (a < 10 meteorological stations-based assessment).

### Temporal rainfall trends at national and sub-national levels

Results from the Man-Kendall test and Sen’s slope estimator showed a decreasing monotonic trend for Pakistan overall, as well as in all provinces, as indicated by the negative coefficient values (Table 3). The Kendall coefficient shows the highest downward trend for Sindh province (coefficient = − 2.28), which is statistically significant at *p* = 0.05. Punjab was the second highest downward trend in the province with a coefficient value of − 1.54; however, this downward trend was not statistically significant. Notably, Sen’s slope estimator showed that the highest decreasing trend in rainfall per year was observed in Punjab province during the study period with a slope value of − 3, followed by Sindh (coefficient = − 1.568) and Gilgit Baltistan (coefficient = − 1.206). For Pakistan at the national level, the downward trend in rainfall was estimated to be − 1.11 mm/year during the study period. These decreasing trends are in line with the findings of^52^, who noted a decreasing rainfall trend in Central India during the period 1949–2012. Our findings are also similar to that of^23^, who found downward trends in Iran’s southeastern and northwestern regions during 1951–2009. This shows that the precipitation in this South-Asian region has experienced downward trend, which is also reflected as “decreased” in the Fifth Assessment Report by the Inter-Governmental Panel on Climate Change^53^.

**Table 3:** Results for Man-Kendall test and San’s Slope estimator for long-term rainfall trends in Pakistan at national and sub-national levels during 1961–2020 (n = 54).

Time series	Test Z (Kendall Coefficient)	Q (San’s Slope)
Balochistan	− 0.49	− 0.311
Gilgit Baltistan (G&B)	− 0.43	− 1.206
Khyber Pakhtunkhwa (KPK)	− 0.10	− 0.126
Punjab	− 1.54	− 3.000
Sindh	− 2.28*	− 1.568
Pakistan	− 0.30	− 1.117

*The value is statistically significant at *p* = 0.05.

Similar to the long-term decreasing trend, the inter-decadal regime shift assessment also showed statistically significant downward regime shifts for the Sindh and Punjab provinces at 90% confidence (Supplementary Fig. S3). It was found that Punjab experienced two downward regimes; first in 2001 and again in 2011. However, Sindh experienced only one downward regime shift in 2001. It should be noted that the occurrence of such downward regime shifts in the recent decades could lead to near-future drought conditions in these provinces without proper planning measures. In contrast, upward shifts were experienced by Gilgit Baltistan and Khyber Pakhtunkhwa provinces in 2004, while downward regimes were later experienced in 2012 (Supplementary Fig. S3). This upward inter-decadal regime shift contrasts with the downward long-term trend in rainfall for these provinces, thus illustrating the significance of long-term and short-term rainfall assessments. At the national level, Pakistan experienced an overall downward regime shift in rainfall (in 2012).

Sindh province is among the most severely drought-affected provinces in Pakistan (see https://bit.ly/36PuUTo). Based on the present analysis, the lower rainfall determined for most parts of the province (i.e., Rohri, Sukkar, Larkana, and Jacobabad, Fig. 5), as well as the statistically significant long-term decrease (Table 3) and inter-decadal downward regime shift in the rainfall (Supplementary Fig. S3), could further stress the region in terms of drought and water availability, affecting millions of people in the near future. Similarly, the regions in southern Punjab (i.e., Bahawalpur, D.G. Khan, and Khanpur) were also found to receive very little rainfall (Fig. 5), thus a decreasing trend in the rainfall could potentially have severe impacts on agriculture as well as water resources of the province. If the management of dams and reservoirs in Pakistan could be improved, nearly 145 million-acre feet water could be conserved annually, based on rainfall observed each year during the monsoon season. The results presented here reflect the need for better water resources management in the Punjab and Sindh provinces, which have the highest populations and are the largest contributors to Pakistan’s total agricultural yield as well as the national Gross Domestic Product.

## Conclusions

In the context of environmental planning and management, this study bridges the existing knowledge gap regarding the spatial distribution and patterns as well as temporal trends (long- and short-term) of rainfall in Pakistan during the past half-century. For spatial assessment of rainfall, we produced the first continuous normal rainfall dataset (spatial resolution of 11 km) using the most optimal interpolation method. For this purpose, 27 interpolation techniques under two broader interpolation categories (i.e., deterministic and stochastic/geo-statistical) were rigorously compared using an AI, which was computed based on multiple cross-validation evaluation criteria. It was concluded that the EBKRP approach is the most optimal method (AI-6 = 0.9425) to interpolate rainfall in Pakistan and should be adopted in the future for national-scale rainfall interpolations. Based on subsequent EBKRP analysis, a spatial variation in the rainfall was identified from south to north across Pakistan, while the highest rainfall was estimated for Malam Jaba in KPK province (~ 1700 mm), and the lowest rainfall was estimated at Nok-kundi in Balochistan province (~ 50 mm). The temporal analysis of rainfall in Pakistan shows an overall decreasing trend at the national and subnational levels. Sindh province experienced a statistically significant decreasing trend in rainfall during the study period, and a recent downward regime shift in rainfall was detected (90% confidence). Similarly, the highest decreasing rate in the rainfall was estimated for Punjab province (-3 mm/year), which is Pakistan’s most populated province and is the largest contributor to the national GDP. The identified decreasing rainfall could escalate the likelihood of droughts, thereby affecting the agricultural sector in an agrarian country already prone to the effects of global warming.

The normal annual rainfall map produced in this study at the national level could be used as an input to hydrological models, integrated to assess flood risk, and research related to agriculture such as crop yield sensitivity to climate change. In Addition, the data could be used in further assessments contributing towards understanding the hydrological processes in Pakistan and aiding in water resources planning and management. Such studies could be carried out in the future. This can provide broader opportunities to reduce the baseline water stress. Additionally, the AI used in this study could also be calculated at different subnational scales to produce similar products at the provincial or district levels in order to produce higher-resolution studies in Pakistan, and beyond. Moreover, the comparison approach presented here is applicable to compare the interpolation methods in diverse scientific areas such as climatology, earth sciences, environmental sciences, and epidemiology, reflecting that the application of the proposed AI is substantially broad. Additionally, as two different wind systems (i.e., Monsoon in summer and Westerly wind system in winter season) are primarily responsible for rainfall in Pakistan, it would be interesting to see, via approach presented here, if the optimal interpolation method for these two systems vary. This could be a potential question for future research.

The authors do acknowledge the limitations of the study at this point. Firstly, the data to compute total annual rainfall for the sake of normal rainfall distribution are mean monthly observations. The use of daily data could result in more comprehensive information and may allow capturing further in-depth details (i.e., extremes) in the temporal analysis. However, this might increase the computational and time costs significantly if analyzed on such large spatial and temporal scales. Similarly, the distribution of rain gauges is not uniform across the study area resulting in sparse gauges in some areas as compared to others (i.e., Balochistan and Northern areas, respectively). No doubt that the EBKRP performs well to predict the rainfall at unknown points, the installation of more observation stations in the regions with sparse distribution of rain gauges is recommended in the context of comprehensive data collection.

## Supplementary Information

Supplementary Information

## Data Availability

The data on rainfall observations for all stations in Pakistan are available online (http://www.pmd.gov.pk/), while the data for the digital elevation model (DEM) used in this study are also available online (https://earthexplorer.usgs.gov/). The final normal rainfall distribution gridded data based on the EBKRP method is available from the corresponding authors upon request.
