# Supplementary material for: Spatial–temporal characterization of rainfall in Pakistan during the past half-century (1961–2020)
Source: Sci Rep. 2021 Mar 25;11:6935. doi: 10.1038/s41598-021-86412-x (PMC7994564; doi:10.1038/s41598-021-86412-x)
Supplement: Supplementary file 1 — Supplementary Information [file 41598_2021_86412_MOESM1_ESM.docx]

**Supplementary Materials for**

**Spatial-Temporal Characterization of Rainfall in Pakistan during the past Half-Century (1961–2020)**

**Ghaffar Ali ^a*^, Muhammad Sajjad ^b, c,*^, Shamsa Kanwal ^d^, Tingyin Xiao ^e^, Shoaib Khalid ^f^, Fariha Shoaib ^f^ , Hafiza Nayab Gul ^g^**

^a^ College of Management, Shenzhen University, Nanhai Ave. 3688, Shenzhen, China. (518060)

^b^ Guy Carpenter Asia-Pacific Climate Impact Centre, School of Energy and Environment, City University of Hong Kong, Hong Kong (SAR)

^c^ Department of Civil and Environmental Engineering, Princeton University, NJ-USA. (08544)

^d^ Department of Land Surveying and Geo-Informatics, The Hong Kong Polytechnic University, Hong Kong SAR.

^e^ Center for Policy Research on Energy and the Environment, Princeton University, NJ-USA. (08544).

^f^ Department of Geography, Government College University Faisalabad, Pakistan.

^g^ School of Geographic Sciences, East China Normal University, Shanghai, PR China.

*Correspondence emails: ([mah.sajjad@hotmail.com](mailto:mah.sajjad@hotmail.com)) and ([ghafar.gs@gmail.com](mailto:ghafar.gs@gmail.com))


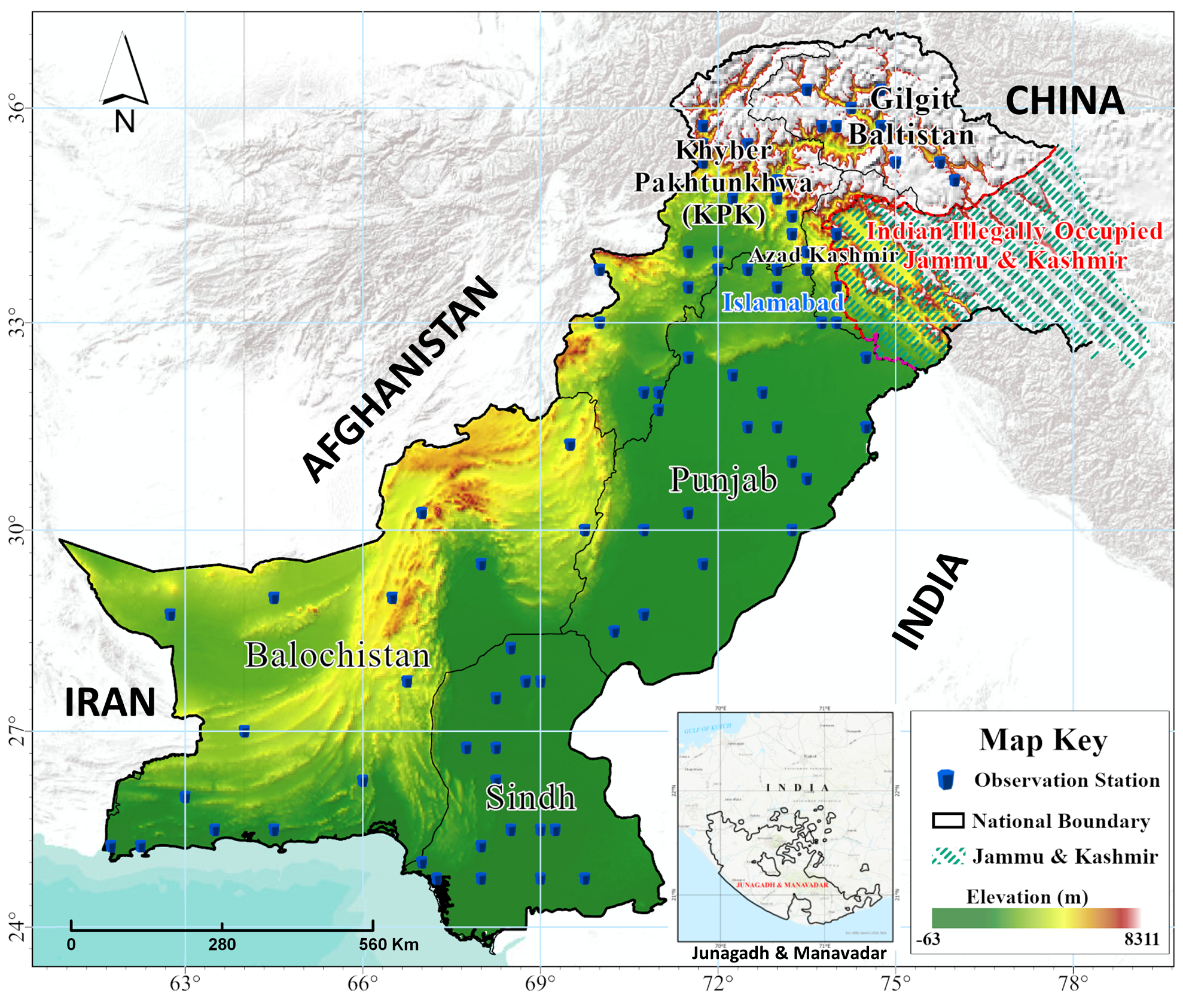


Supplementary Fig. S1: Study area map showing the geographic distribution of rain gauges (n = 82) in Pakistan and the digital elevation model (30-m resolution) showing the elevation (m) of different regions throughout the study area. The study area map is designed *by a co-author M.S. using ArcGIS Pro (Version 2.7) from the Environmental Systems Research Institute (ESRI), available at* [*www.esri.com*](http://www.esri.com)*.* *The Digital Elevation Model used as a basemap is retrieved from the United States Geological Survey data platform; available at* [*www.usgs.gov*](http://www.usgs.gov)*.*


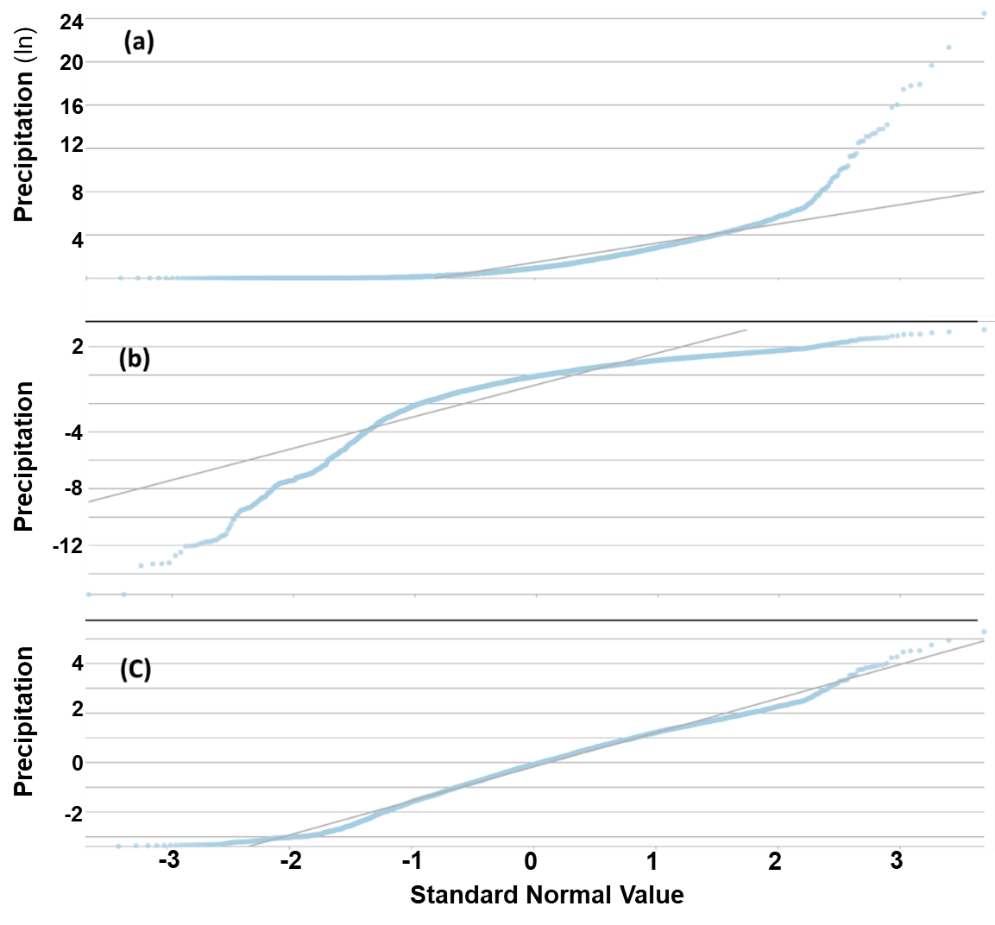


Supplementary Fig. S2: QQ plot for the data: a) original data, b) log-transformed data, and c) Box-Cox transformed data with λ = 0.29. The values for precipitation on the y-axis of panels b and c are standardized. *The plots are made using ArcGIS Pro (Version 2.7) from the Environmental Systems Research Institute (ESRI) by a co-author M.S., available at* [*www.esri.com*](http://www.esri.com)*.*


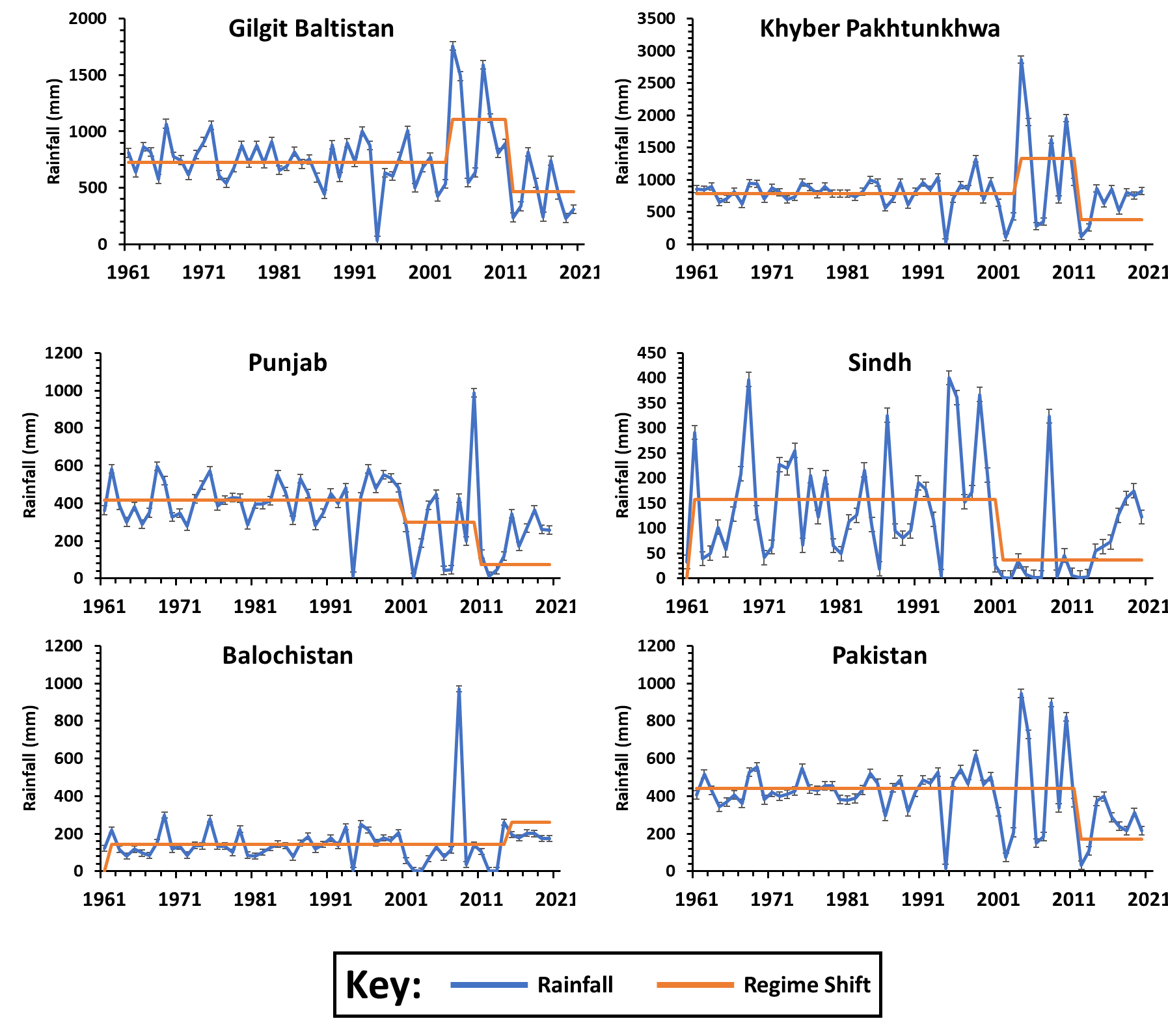


Supplementary Fig. S3: Temporal rainfall in Pakistan and regime shifts detection based on Student’s t-test algorithm with 90% confidence. The values at x-axis show years and the values at y-axis show rainfall in mm. *The plots are made using Microsoft Excel software (Version 365) by a co-author M.S., available at* [*www.microsoft.com*](http://www.microsoft.com)*.*
